# Cardiovascular Hospitalizations Burden Following Septal Myectomy for Obstructive Hypertrophic Cardiomyopathy

**DOI:** 10.1161/JAHA.124.040655

**Published:** 2025-07-14

**Authors:** Ahmed Altibi, Miriam Elman, Hailey Volk, Mohammad Alqabani, Michael Butzner, Howard Song, Ahmad Masri

**Affiliations:** ^1^ Hypertrophic Cardiomyopathy Center, Division of Cardiology Knight Cardiovascular Institute, Oregon Health and Science University Portland OR USA; ^2^ Department of Cardiovascular Medicine Yale University School of Medicine New Haven CT USA; ^3^ School of Public Health Oregon Health & Science University‐Portland State University Portland OR USA; ^4^ Cytokinetics, Incorporated South San Francisco CA USA; ^5^ Division of Cardiothoracic Surgery Oregon Health and Science University Portland OR USA

**Keywords:** atrial fibrillation, cardiovascular hospitalization, hypertrophic cardiomyopathy, septal myectomy, Cardiomyopathy

## Abstract

**Background:**

Longer‐term morbidity post septal myectomy (SM) in obstructive hypertrophic cardiomyopathy has not been well characterized at a national level. We aimed to investigate the nonfatal longer‐term post‐SM outcomes from a national all‐payer individual‐level claims.

**Methods:**

The Symphony Health Claims database (2016–2021) was analyzed to identify all adult patients with obstructive hypertrophic cardiomyopathy who underwent SM in the United States and had at least 1 claim within 120 days before SM. The primary outcome was cardiovascular hospitalizations (CVH) starting >30 days post‐SM.

**Results:**

A total of 5324 patient (median age 62.0 [52.0–70.0], 53.2% female, 70% commercial insurance) underwent SM and 95.8% were followed >30 days post SM. During 2.7 (1.2–4.2) years median follow‐up, CVH occurred in 46.7% (80% of CVH within 16 months of SM). CVH for new atrial fibrillation/flutter was 25.4%, ventricular arrhythmias 9.7%, syncope 9.3%, myocardial infarction 5.2%, cardiac arrest 1.6%, ventricular septal defect 0.9%, and need for advanced heart failure therapy 0.6%. Repeat SM was required in 43 patients (0.8%). The strongest predictors of CVH post SM were presence of an implantable cardioverter‐defibrillator at baseline (adjusted odds ratio [aOR], 1.72 [95% CI, 1.50–1.97], *P*<0.001), chronic obstructive pulmonary disease (aOR, 1.65 [95% CI, 1.44–1.89], *P*<0.001), and chronic kidney disease (aOR, 1.45 [95% CI, 1.26–1.66], *P*<0.001).

**Conclusions:**

Over a 3‐year period, SM for obstructive hypertrophic cardiomyopathy was associated with a high burden of CVH. Investigating the drivers of these events and strategies to mitigate the high incidence of intermediate and long‐term nonfatal complications post SM will help improve the care of patients with obstructive hypertrophic cardiomyopathy.

**Nonstandard Abbreviations and Acronyms**
AFL: atrial flutter
CVH: cardiovascular hospitalizations
HCM: hypertrophic cardiomyopathy
oHCM: obstructive hypertrophic cardiomyopathy
SM: septal myectomy

**Clinical Perspective**

**What Is New?**

- In this national cohort study of 5324 patients with obstructive hypertrophic cardiomyopathy undergoing septal myectomy, there was a high burden of cardiovascular hospitalizations, affecting 46.7% of patients over a median 2.7‐year follow‐up, with heart failure and new‐onset atrial arrhythmias as primary drivers.

**What Are the Clinical Implications?**

- The high rate of cardiovascular hospitalizations post septal myectomy, particularly within the first year, underscores the need for enhanced postoperative monitoring and management strategies targeting heart failure and atrial arrhythmias to reduce morbidity in patients with obstructive hypertrophic cardiomyopathy, even after successful relief of left ventricular outflow tract obstruction.
- With the known low mortality in patients with obstructive hypertrophic cardiomyopathy, understanding the nonfatal outcomes post septal myectomy enables a better understanding of the comparative performance of septal myectomy as compared with other medical and catheter‐based interventions.

Left ventricular outflow tract (LVOT) obstruction is present in about 70% of patients with hypertrophic cardiomyopathy (HCM) and is associated with worse outcomes and higher mortality.^1^, ^2^ Over the past 6 decades, septal myectomy (SM) has been the mainstay for abolishing LVOT obstruction in patients who remain symptomatic despite maximal medical therapy.^3^

Numerous studies have demonstrated the efficacy of SM in relieving LVOT obstruction, improving hemodynamics, and quality of life after surgery. In addition, patients with oHCM commonly have additional structural abnormalities of the submitral valve as part of the obstructive HCM (oHCM) disease complex or intrinsic mitral or aortic valvular disease, especially in the aging population with HCM.^4^, ^5^ In these cases, SM offers the added benefit of correcting such valvular abnormalities via concomitant mitral valve/submitral apparatus repair, mitral valve replacement, or aortic valve replacement, at the expense of increasing surgical complexity.^6^, ^7^

Surgically, SM remains a complex procedure, with a steep learning curve, that is best performed in the hands of experienced operators at centers with high procedural volume.^8^ Despite that, real‐world data have shown SM to have a very low (<1%–5%) operative mortality and excellent short‐term procedural outcomes.^9^, ^10^ Although several studies characterized midterm and long‐term outcomes of SM, contemporary data are limited to reports from a single center or few centers with a focus on short‐term outcomes and all‐cause mortality.^11^, ^12^ In a single‐center study with the longest follow‐up duration >10 years, survival following SM in 139 patients was 93% and remission from advanced heart failure (HF) to New York Heart Association Class I–II was sustained in 92% of patients; suggesting that SM could provide long‐term symptomatic and survival benefit.^13^ With low mortality, investigating the nonfatal outcomes and morbidity post SM in a national cohort is vital to understanding the natural history of disease after this intervention. As such, the goals of this study are to (1) report on characteristics and short‐term outcomes of SM in a payer‐agnostic national cohort of all‐comers with oHCM, and (2) explore nonfatal complications following SM, focusing on cardiovascular hospitalization (CVH) and its predictors.

## METHODS

Access to the database can be obtained through the Symphony database and will not be made available by the authors.

### Database

An observational study using individual‐patient claims data from Symphony Health database. These data are payer agnostic and include individual‐level health care claims for >280 million US‐based commercial and Medicare Advantage enrollees from pharmacy fill, diagnosis, procedure, and surgery claims. The study period was January 1, 2016 to November 20, 2021. Adult patients with oHCM diagnosis who underwent SM (2016–2021) were included. The present study was deemed exempt by Oregon Health & Science University Institutional Review Board and informed consent not required as the study used deidentified data from administrative databases.

### Cohort Selection

Adult patients with HCM were identified from diagnostic claims using *International Classification of Diseases, Tenth Revision* (*ICD‐10*) codes (I42.1 and I42.2) with a qualifying SM procedure during the study period (Table S1). Qualifying SM procedures were identified using Current Procedural Terminology and *ICD‐10‐Procedure Coding System* procedures codes, respectively (Table S1). Each patient contributed a single index procedure. Baseline characteristics were derived from claims codes in the 90 days before SM. Exclusion criteria included patients <18 years old, patients without claims within 120 days before SM, and patients without 30 days of follow‐up (procedures performed November 2021). The flow diagram for the patient selection process is depicted in Figure S1.

### Defining Periods for Baseline Variables, Acute, and Longer‐Term Complications

For acute diagnoses, procedures, and surgeries, we defined the period before the index procedure as the 4 to 93 days before the SM or concomitant procedure, whichever came first chronologically. Comorbidities were included if they appeared before SM, including diabetes, obesity, hyperlipidemia, hypertension, tobacco use, chronic obstructive pulmonary disease (COPD), and chronic kidney disease (CKD). We defined the required follow‐up period after the index procedure as the −3 to 27 days post SM or concomitant procedure, whichever claim came first chronologically, except for repeat SM, coronary angiograms, and percutaneous coronary interventions. For these procedures, follow‐up was assessed in the 3 to 30 days post‐index or ‐concomitant procedure. The rationale for including up to 3 days before SM relates to the slight variation in the timeliness and exact date of a claim occurring in conjunction with SM. In addition, some patients are admitted right before SM. Short‐term outcomes are defined as those occurring within 30 days of index procedure, and long‐term outcomes are those occurring beyond the 30‐day period up to the last claim for each patient.

### Study Outcomes

The primary outcome of the study was CVH during follow‐up period. Patients without events were censored at their last claim (ie, appearance) in the database. CVH was defined as the composite of adverse cardiovascular events requiring hospitalization following SM as detailed subsequently. Secondary outcomes were (1) in‐hospital events post SM (cardiac arrest, cerebral infarction, anoxic brain damage, cardiopulmonary resuscitation, emergency intubation, tracheostomy, hemothorax, new dialysis, ventricular septal defect, and repeat tracheostomy), and (2) long‐term outcomes following SM (new‐onset atrial fibrillation [AF] or atrial flutter [AFL], pacemaker or implantable cardioverter‐defibrillator) implantation, ventricular arrhythmia, myocardial infarction, stroke, and need for advanced HF therapy. The Current Procedural Terminology and *ICD‐10* codes used to identify outcomes of interest are summarized in Table S1. To confirm the findings, manual code review was performed for every claim code that occurred ≥5 instances. The claims data do not provide death data and, as such, cannot be analyzed. Death is not coded in the Symphony database, and as such we did not use survival as an outcome. The following definitions were used for outcomes:

1. CVH: hospital, inpatient, emergency department, or nursing facility interaction based on diagnosis or procedural claims present within 7 days of the event, and at least 30 days post SM. CVH was defined as a composite of cardiac arrest, ventricular fibrillation, ventricular tachycardia, myocardial infarction, HF, stroke, infective endocarditis, syncope, new hospital‐associated AF/AFL, new dialysis, heart transplant, and ventricular assist device implantation.
2. New onset AF/AFL defined as the presence of a diagnosis claim for AF/AFL after the 30 days post SM without the presence of a code at baseline (pre SM and within 30 days post SM).
3. New dialysis was defined as the presence of a diagnosis or procedural claim for dialysis after the 30 days post SM without the presence of a code at baseline or 30‐day period post SM.

### Statistical Analysis

Descriptive analyses were conducted for study variables. The continuous variable, age, was reported as median with interquartile range and the remaining categorical variables were reported as percentages with frequencies. Models were constructed to identify predictors of CVH post SM. Candidate predictors were selected a priori as risk factors based on previous studies and clinical input. These variables included: age, sex, smoking status, diabetes, obesity, hypertension, COPD, CKD, mitral, aortic, or tricuspid valve disease, AF or AFL, presence of a pacemaker or implantable cardioverter‐defibrillator.

We assessed univariable models for the candidate risk factors with each outcome. Subsequently, we performed multivariable logistic regression, where we fit the full model for each outcome including all candidate risk factors, then performed variable selection using backwards elimination based on the Akaike information criterion (corresponding to a significance level of *P*=0.157) for a stopping criterion as recommended by Heinze et al. (2018).^14^ Results from univariable, full, and selected models are presented as odds ratios (OR) with corresponding 95% CIs. The Kaplan–Meier methods were used to plot the incidence of CVH and new‐onset AF/AFL over the follow‐up period. Data management and analyses were performed using SAS v9.4 (SAS Institute, Cary, NC) and R 4.2.0 (R Core Team, Vienna, Austria).

## RESULTS

Of 5673 unique individuals who underwent SM, we excluded 201 patients <18 years old (Flow Diagram, Figure S1). To allow for a 30‐day follow‐up period following the index procedure, we further excluded 41 patients with index procedures performed in November 2021. Finally, 107 patients who did not have a diagnosis code in the 120 days before their SM were removed.

### Baseline Characteristics

A total of 5324 patients (median age 62.0 [interquartile range, 52.0–70.0], 53.2% were female) were included in the study. The median follow‐up duration was 2.7 (interquartile range, 1.2–4.2) years. Baseline characteristics and comorbidities of the study cohort are shown in Table 1. The most prevalent comorbidities were hypertension (n=2997, 56.3%), hyperlipidemia (n=1793, 33.7%), and coronary artery disease (n=1617, 30.4%). About 17.0% (n=905) had prior diagnosis of AF/AFL, 8.1% (n=432) had implantable cardioverter‐defibrillator, and 3.2% (n=172) had a permanent pacemaker.

**Table 1:** Selected Comorbidities and Short‐Term Complications Following SM

Diagnosis	Septal myectomy
Baseline comorbidities	(n=5324)
Age, y, median (interquartile range)	62.0 (52.0–70.0)
Female sex, n (%)	2849 (53.5)
Hypertension	2997 (56.3)
Hyperlipidemia	1793 (33.7)
Coronary artery disease	1617 (30.4)
Obesity	996 (18.7)
Any atrial fibrillation/flutter	905 (17.0)
Diabetes	752 (14.1)
Presence of ICD	432 (8.1)
Chronic kidney disease	415 (7.8)
Chronic obstructive pulmonary disease	384 (7.2)
Presence of cardiac pacemaker	172 (3.22)
Pericardial effusion	92 (1.7)
Cardiac arrest	41 (0.8)
End‐stage renal disease on dialysis	24 (0.5)
Nonrheumatic mitral valve disorder	2109 (39.6)
Nonrheumatic aortic valve disorder	1295 (24.3)
Nonrheumatic tricuspid valve disorder	223 (4.2)
Complications*	
New atrial fibrillation/flutter	1498 (28.1)
New pacemaker/ICD†	756 (14.2)
Acute kidney failure	730 (13.7)
Ventricular fibrillation/tachycardia	613 (11.5)
Heart block and new pacemaker/ICD	526 (9.9)
Cardiogenic/unspecified shock†	463 (8.7)
Blood transfusion†	289 (5.4)
Cardioversion	235 (4.4)
Coronary angiogram/percutaneous coronary intervention	200 (3.8)
Ventricular septal defect	173 (3.2)
Cardiac arrest	125 (2.3)
Stroke	116 (2.2)
Emergency intubation†	100 (1.9)
Tracheostomy	92 (1.7)
New dialysis	78 (1.5)
Extracorporeal membrane oxygenation†	55 (1.0)
Ventricular septal defect repair	49 (0.9)
Hemothorax	36 (0.6)
Anoxic brain damage	8 (0.1)
Repeat septal myectomy	43 (0.8)
Transient ischemic attack	39 (0.7)

ICD indicates internal cardioverter‐defibrillator; and SM, septal myectomy.

* Complications assessed in 0 to 30 days after the index procedure except where otherwise indicated.

^†^ Assessed in the 1 to 30 days post index procedure.

Valvular heart diseases were prevalent with 39.6% (n=2109) having nonrheumatic mitral valve disorder and 24.3% (n=1295) having nonrheumatic aortic valve disorder. Concomitant mitral valve repair occurred in 33.4% (n=1778), mitral valve replacement in 13.2% (n=703), papillary muscle intervention in 11.8% (n=628), and left atrial appendage clipping/excision in 11.8% (n=628). There were 48 (0.9%) patients without diagnosis, procedure, surgery, or prescription claims within 30 days and 1 year post SM.

#### Inpatient and Short‐Term Complications Following SM

We assessed short‐term complications (0–30 days) after SM as an index procedure. New AF/AFL occurred in 1498 (28.1%) patients. Heart block requiring pacemaker/defibrillator implantation occurred in 526 (9.9%) patients. Cardiogenic/unspecified shock post SM occurred in 463 (8.6%) patients. Ventricular septal defect occurred in 173 (3.2%) patients, but only 49 (0.9%) had ventricular septal defect repair performed. Repeat SM within 30 days was required in 43 (0.8%) patients. Short‐term complications following SM are summarized in Table 1. Subsequently we stratified the complications by age (<65 years versus ≥65 years), Table S2. There were no systematic differences in the rate of complications, except for acute kidney injury and any AF/AFL in patients ≥65 years, whereas ventricular arrhythmias were more common in patients <65 years. In the overall cohort, the strongest predictors for inpatient complications post SM were prior AF/AFL (adjusted OR [aOR], 1.53 [95% CI, 1.23–1.89], *P*<0.001), CKD (aOR 1.54 [95% CI, 1.16–2.03], *P*<0.01), and COPD (aOR 1.46 [95% CI, 1.08–1.95], *P*=0.01) as detailed in Table 2.

**Table 2:** Predictors of In‐Hospital Complications Following SM

Variable	aOR (95% CI)	*P* value
Female sex	1.17 (0.98–1.40)	0.09
Obesity	0.85 (0.67–1.06)	0.16
Hypertension	1.21 (1.00–1.47)	0.048
Chronic obstructive pulmonary disease	1.46 (1.08–1.95)	0.01
Prior atrial fibrillation/atrial flutter	1.53 (1.23–1.89)	<0.001
Chronic kidney disease	1.54 (1.16–2.03)	0.002
Age	1.00 (0.99–1.01)	0.91
Diabetes	0.95 (0.74–1.21)	0.68
Tobacco smoking	0.94 (0.74–1.21)	0.60
Mitral valve disease	1.06 (0.88–1.27)	0.53
Aortic valve disease	1.21 (0.99–1.48)	0.06
Tricuspid valve disease	0.89 (0.57–1.33)	0.57
Implantable cardioverter‐defibrillator	1.22 (0.89–1.65)	0.19
Pacemaker	1.29 (0.82–1.91)	0.25

aOR indicates adjusted odds ratio; and SM, septal myectomy.

#### Long‐Term Cardiovascular Hospitalizations Following SM

**Figure 1:**
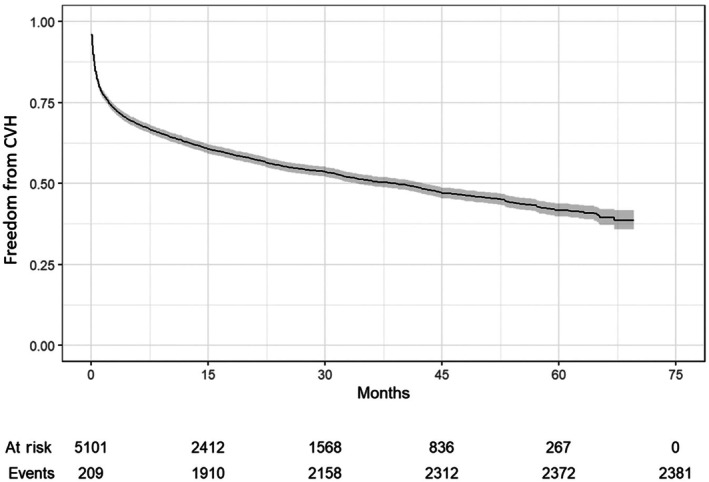
CVH following SM. Kaplan–Meier curve showing cardiovascular hospitalization over the follow‐up period after 30 days of septal myectomy. CVH indicates cardiovascular hospitalization.

A total of 5101 (95.8% of the overall cohort) had follow‐up beyond 30 days post SM. Over a median of 2.7 years (interquartile range, 1.2–4.2), 2381 (46.7%) CVH occurred (Figure 1) with 80% of hospitalizations occurring within 16 months post SM, Figure 1. On multivariable regression analysis, the strongest predictors for CVH following SM were presence of implantable cardioverter‐defibrillator at baseline (aOR, 1.72 [95% CI, 1.50–1.97], *P*<0.001), pacemaker (aOR, 1.41 [95% CI, 1.16–1.72], *P*<0.0001), COPD (aOR, 1.65 [95% CI, 1.44–1.89], *P*<0.001), and CKD (aOR, 1.45 [95% CI, 1.26–1.66], *P*<0.001), Table 3. The distribution of predictors for CVH is shown in Table S3. Patients who had CVH post SM were more likely to have hypertension, COPD, CKD, prior pacemaker/implantable cardioverter‐defibrillator implant, and diabetes (Table S3).

**Table 3:** Predictors of Long‐Term Cardiovascular Hospitalizations in Patients Undergoing Myectomy

Predictors	Univariable model		Full multivariable model		Selected model*	
	OR (95% CI)	*P* value	aOR (95% CI)	*P* value	aOR (95% CI)	*P* value
Age	1.01 (1.01–1.01)	<0.001	1.01 (1.00–1.01)	0.0023	1.01 (1.00–1.01)	0.002
Female sex	1.21 (1.11–1.31)	<0.001	1.12 (1.03–1.22)	0.0076	1.12 (1.03–1.22)	0.01
Diabetes	1.39 (1.24–1.54)	<0.001	1.16 (1.04–1.30)	0.0080	1.16 (1.04–1.30)	0.01
Obesity	1.37 (1.24–1.51)	<0.001	1.20 (1.08–1.33)	<0.0001	1.20 (1.08–1.33)	<0.001
Hypertension	1.48 (1.37–1.61)	<0.001	1.24 (1.13–1.36)	<0.0001	1.24 (1.14–1.36)	<0.001
Chronic obstructive pulmonary disease	1.91 (1.67–2.18)	<0.001	1.64 (1.43–1.89)	<0.0001	1.65 (1.44–1.89)	<0.001
Nonrheumatic mitral valve disorder	1.30 (1.19–1.41)	<0.001	1.18 (1.08–1.28)	<0.0001	1.19 (1.09–1.29)	<0.001
Nonrheumatic aortic valve disorder	1.22 (1.12–1.34)	<0.001	1.09 (0.99–1.20)	0.0712	1.10 (1.00–1.21)	0.06
Chronic kidney disease	1.66 (1.45–1.90)	<0.001	1.45 (1.26–1.66)	<0.0001	1.45 (1.26–1.66)	<0.001
Implantable cardioverter‐defibrillator	1.68 (1.48–1.91)	<0.001	1.72 (1.50–1.97)	<0.0001	1.72 (1.50–1.97)	<0.001
Pacemaker	1.82 (1.51–2.19)	<0.001	1.41 (1.16–1.71)	<0.0001	1.41 (1.16–1.72)	<0.001
Tobacco use	1.18 (1.07–1.31)	<0.001	1.01 (0.91–1.12)	0.8412		
Nonrheumatic tricuspid valve disorder	1.40 (1.16–1.68)	<0.001	1.11 (0.92–1.34)	0.2883		

aOR indicates adjusted odds ratio;and OR, odds ratio.

* Model selected by backwards elimination based on Akaike information criterion.

**Figure 2:**
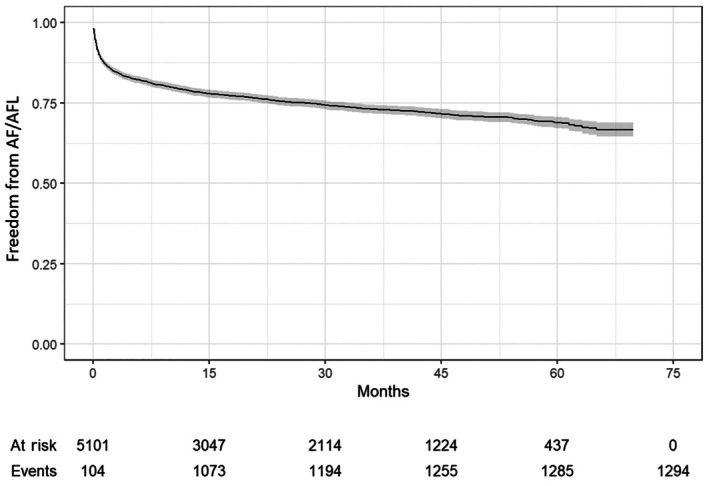
Atrial arrhythmias following SM. Kaplan–Meier curve showing new atrial fibrillation/flutter over the follow‐up period after 30 days of septal myectomy. AF/AFL indicates atrial fibrillation/atrial flutter.

The specific causes of CVH post SM are shown in Table 4. HF was responsible for the highest number of CVH at 1446 (28.3%), and new‐onset AF/AFL occurred in 1294 patients (25.4%), Figure 2. Ventricular arrhythmias were responsible for 9.7% of CVH occurring in 497 patients. Myocardial infarction and stroke occurred in 265 (5.2%) and 202 (4.0%) patients during the follow‐up period.

**Table 4:** Long‐Term Outcomes Following Septal Myectomy in Obstructive Hypertrophic Cardiomyopathy

Outcome	No.=5101	(%)
Cardiovascular hospitalizations	2381	(46.7)
Heart failure	1446	(28.3)
New atrial fibrillation/flutter	1294	(25.4)
Ventricular fibrillation/tachycardia	497	(9.7)
Syncope	473	(9.3)
Myocardial infarction	265	(5.2)
Cardioversion	235	(4.6)
Stroke	202	(4.0)
Cardiac arrest	81	(1.6)
Infective endocarditis	82	(1.6)
Dialysis	51	(1.0)
Ventricular assist device	18	(0.4)
Heart transplant	11	(0.2)

The majority of new‐onset AF/AFL occurred during the first 15 months following SM as shown in Figure 2. The strongest predictors for new‐onset AF/AFL were COPD (aOR, 1.40 [95% CI, 1.15–1.69], *P*<0.001), CKD (aOR, 1.21 [95% CI, 1.01–1.47], *P*=0.046), and age (aOR, 1.03 [95% CI, 1.02–1.03], *P*<0.001), Table S4. The distribution of predictors for new‐onset AF/AFL is shown in Table S3. Patients who developed new‐onset AF/AFL post SM were more likely to have hypertension, COPD, and CKD at baseline (Table S3).

## DISCUSSION

In this cohort study from a large nationally representative all‐payer individual‐level claims database, we investigated the nonfatal cardiovascular outcomes of adult patients with oHCM who underwent SM between 2016 and 2021 in the United States. We found that (1) incidence of CVH following SM was substantial occurring in almost half of patients following SM; (2) the majority of CVH following SM occurred within the first year and a half postoperatively; (3) HF was a primary driver for CVH following SM occurring in at least one fourth of patients; and (4) new‐onset AF/AFL was prevalent following SM, occurring in one fourth of patients.

Historically, patients with HCM were estimated to have an annual mortality rate of 3% to 6% with poor outcomes primarily attributed to fatal arrhythmias and progressive HF.^15^ However, contemporary data have shown the improved survival with contemporary management, and symptomatic significant LVOT obstruction should be addressed to prevent HF progression and hospitalizations.^16^, ^17^ Despite the substantial reduction in annual HCM‐related mortality to <1% per year in most contemporary cohorts, at least 1 in 15 patients with HCm (6%–7%) develops advanced HF marked by having a medically refractory disease with severe functional impairment.^18^

SM is a highly effective, low‐risk surgery in the hands of experienced surgeons, used to abolish the LVOT obstruction and relief symptoms.^8^ SM improves survival as well compared with ongoing symptomatic LVOT obstruction. In 289 patients with oHCM who underwent SM, Ommen et al. reported a 5‐ and 10‐year survival after SM of 96% and 83%, respectively, compared with 79% and 61% respectively, in nonoperated patients with LVOT obstruction.^19^ The majority of studies in SM are focused on short‐term outcomes and long‐term survival, which are known to be excellent in centers with expertise performing SM in high volumes. Additional studies that reported on HF and clinical outcomes are typically from single‐center studies.^20^ There has not been a comprehensive multicenter longer‐term systematic assessment of nonfatal CVH in this population, which is the primary cause of morbidity in this population.

In this analysis of 5324 individual patients with oHCM who underwent SM, and over a median follow‐up of 2.7 years, there was a significant burden of nonfatal CVH, which occurred in up to half patients following SM with the majority occurring within the first year and a half after surgery. These findings do not include the immediate 30 days post SM, which is expected to be the highest risk period. CVH appeared to be primarily driven by HF and new‐onset AF/AFL. In the majority of patients with HCM, LVOT obstruction results from systolic anterior motion of the mitral valve, which leads to severely elevated LV pressure and mitral regurgitation.^15^ In those patients, extended SM, performed to abolish the LVOT gradient and reduce mitral regurgitation, improves exercise capacity, reduces HF symptoms, and improves survival in observational cohort studies.^15^, ^16^, ^18^ However, a subset of patients with oHCM post SM continue to be burdened by HF symptoms related to residual hypercontractility, impaired relaxation, diastolic dysfunction and increased wall stress. Such patients behave similar to patients with symptomatic nonobstructive HCM.^15^ These findings are typically more common in patients with significant hypertrophy in the mid‐ to distal septum. In addition, a minority of patients with HCM develop progressive end‐stage HF in the absence of LVOT obstruction and are typically referred for heart transplantation.^15^, ^16^, ^18^ In our cohort, at least one fourth of patients had a CVH related to HF in the 2.7 years follow‐up period after SM. However, the structure of the claim data set limits the ability to investigate further why patients developed HF.

AF/AFL occurred frequently and was associated with 25.4% of CVH post SM, even after excluding the immediate postoperative period. AF is the most common sustained arrhythmia in patients with HCM and is an important cause of morbidity, impaired quality of life, embolic stroke, and mortality. Prior single‐center studies have shown somewhat similar AF/AFL rate of 20% to 25%, but over a 5‐year period.^21^, ^22^ In 1 study, in‐hospital postoperative AF was associated with worse clinical outcomes after SM.^23^ In another cohort study of 494 patients with oHCM undergoing SM, post‐operative new‐onset AF was associated with 2‐fold increase in the risk of adverse cardiac events, which was a similar risk to those with preexisting AF preoperatively.^23^ In our multivariable analysis, preexisting AF/AFL was the strongest predictor for in‐hospital complications post SM (aOR, 1.54 [95% CI, 1.26–1.89], *P*<0.001). It remains unclear why patients with oHCM post SM have a high incidence of AF even after the relief of LVOT obstruction. It is likely that AF is a surrogate for the degree of atrial myopathy, residual increased LV and atrial filling pressures, and impaired relaxation in this population.

The impact of SM on AF incidence and burden on the long‐term has not been systematically evaluated in a prospective fashion. SM has been shown to reduce the likelihood of new‐onset AF.^22^ In a consecutive cohort with oHCM (n=1625) without AF at baseline, the risk of new‐onset AF was 30% lower in patients with oHCM after SM than that for patients with nonoperated oHCM (17% versus 26% at 10 years, *P*=0.04).^22^ Notably, the risk for new‐onset AF after SM was similar, not lower, to that for the subgroup with nonobstructive HCM at 10 years.^22^

Additionally, in our cohort, the burden of new conduction abnormalities post SM was substantial as 526 patients (n=9.9%) required a pacemaker or defibrillator in the early postoperative period for new‐onset complete heart block. Conduction abnormalities, including left bundle‐branch block, are a known and frequent sequela post SM. New left bundle‐branch blocks were reported to occur in ~40% of patients post SM with no impact on postoperative mortality.^24^ In a single‐center cohort of 2482 patients, the incidence of complete heart block post SM in all‐comers at 2.3%. However, in the subset of patients with preexisting right bundle‐branch block who underwent SM, the risk of complete heart block was greatly increased to ~34% post SM.^24^ Importantly, paced rhythm in oHCM was independently associated with increased mortality in patients with oHCM post SM; therefore, strategies to avoid complete heart block after SM become imperative.^24^

The safety and efficacy of SM in oHCM have rigorously been studied for many decades.^8^ The issue remains that the majority of these studies are from high‐volume highly experienced centers. Prior studies have established an inverse volume–outcome relationship for myectomy, where mortality outcomes pos SM in such high‐volume centers tend to be significantly lower than those from low‐volume centers.^8^ Further, these reports typically focus on isolated SM, which subselects for a simpler group of patients and risks selection bias.^25^ We chose the Symphony database based on the desire to investigate long‐term nonfatal complications following SM in a national cohort that is payer‐agnostic with available individual‐level claims. These analyses are not feasible in any of the major national databases, including the commonly used Healthcare Cost and Utilization Project, which employs survey‐based approaches, and the Center for Medicare and Medicaid Services data sets, which would exclude two thirds of patients with HCM.^26^

### Limitations

First, our analysis relies on a claims database that did not allow for the assessment of structural and hemodynamic changes following SM, such as LVOT gradient, left atrial size, and mitral regurgitation severity, therefore, limiting our interpretation for the mechanisms leading to adverse cardiac events following SM. Second, the Symphony database lacks clinically important information such as HCM phenotype, symptoms, and procedural details. Further, the details of imaging studies (echocardiogram or cardiac magnetic resonance imaging) at baseline or follow‐up are not available to be reported by the database. Third, we are unable to assess mortality post SM in this cohort given that it is not coded in this database. Our analysis focused on nonfatal complications post SM. Nonetheless, we have previously analyzed mortality outcomes of SM in the United States and reported a lower in‐hospital mortality rate of 4% among all comers (n=in 12 065 patients who underwent SM) at the national level. With such low mortality rate associated with SM, investigating nonfatal outcomes, such as CVH and arrhythmia burden, becomes more imperative.^8^, ^25^ Finally, our analysis predated the Food and Drug Administration approval (April 2022) of the first‐in‐class cardiac myosin inhibitors for oHCM. The interplay between cardiac myosin inhibitors and surgical myectomy in this era of emerging novel therapeutics is to be determined in future studies.

## CONCLUSIONS

Over a 3‐year median follow‐up period, SM for oHCM was associated with a high burden of CVHs that were primarily driven by HF and new onset AF/AFL. Investigating the drivers of these events and strategies to mitigate the high incidence of intermediate and long‐term nonfatal complications post SM will help improve the care of patients with oHCM.

## Sources of Funding

Access to the data was under a collaborative research agreement with Cytokinetics, who funded data access only. Otherwise, all the analyses and the project related work were unfunded and independently performed at the Oregon Health and Science University Hypertrophic Cardiomyopathy Center.

## Disclosures

Michael Butzner is a full‐time employee of and holds stock in Cytokinetics, Inc. Howard K. Song serves as a consultant for Edwards Lifesciences and Medtronic. Ahmad Masri reports research grants from Pfizer, Ionis, Attralus, and Cytokinetics and consulting fees from Cytokinetics, BMS, BridgeBio, Pfizer, Ionis, Lexicon, Attralus, Alnylam, Haya, Alexion, Akros, Prothena, BioMarin, AstraZeneca, and Tenaya. Other authors have no disclosures. All authors have seen and approved the final version of the article being submitted.

## Supporting information

Tables S1–S4
Figure S1
