# Supplementary material for: Cardiovascular Hospitalizations Burden Following Septal Myectomy for Obstructive Hypertrophic Cardiomyopathy
Source: J Am Heart Assoc. 2025 Jul 14;14(14):e040655. doi: 10.1161/JAHA.124.040655 (PMC12533672; doi:10.1161/JAHA.124.040655)

SUPPLEMENTAL MATERIAL

Table S1. Qualifying procedure and surgery codes for procedures concomitant to septal myectomy.

Procedure	CPT Codes	ICD-10-PCS Codes
**Hypertrophic Cardiomyopathy**		I42.1 and I42.2
**Septal myectomy**	33416, 33542	02BM0ZZ. 02BL0ZZ, 02TM0ZZ
**Aortic valve replacement**	33405, 33410, 33859, 33860, 33863, 33864, 33870	02BF0ZZ, 02RF08Z, 02RF07Z, 02RF0JZ, 02RW0JZ, 02BX0ZZ, 02QX0ZZ, 02RX0JZ, 02UX08Z, 02UX0JZ
**Surgical Aortic valve replacement**	33391, 33400, 33411, 33390	02QF0ZZ, 02UF08Z, 02UF0JZ
**Mitral valve replacement**		02RG0KZ, 02RG0JZ, 02BG0ZZ, 02RG08Z
**Mitral valve repair**	33422, 33425, 33427, 33430	02NG0ZZ, 02QG0ZZ, 02UG08Z, 02UG0JZ, 02VG0ZZ
**Tricuspid valve replacement**	33465	02QJ0ZZ
**Tricuspid valve repair**	33463, 33464	02UJ0JZ
**LAA clipping**		02570ZK, 02L70CK, 02L70ZK
**LAA excision**		02B70ZK
**Papillary muscle intervention**		02890ZZ, 02BD0ZZ, 02N90ZZ, 02ND0ZZ, 02QD0ZZ, 02R90JZ, 02T90ZZ, 02TD0ZZ, 02B90ZZ
**Atrial fibrillation ablation**	33257, 33258, 33259	
**Pacemaker**	33206, 33208	02H60JZ, 02H63JZ, 02HK0JZ, 02HK3JZ, 02HK3NZ, 0JH606Z
**Implantable cardioverter defibrillator (ICD)**	33249, 93287	02H63KZ, 02HK3KZ, 0JH608Z
**Atrial fibrillation/flutter***		I48.0, I48.1, I48.2, I48.4, I48.91
**Cardiac arrest**		I46.9, I97.120
**Cardiopulmonary resuscitation (CPR)**	92950	

**Transient ischemic attack (TIA)**		G45.9
**Cerebral infarction**		I63.10, I63.411, I63.511, I63.8, I63.9
**Anoxic brain damage**		G93.1
**Hemothorax**		J94.2
**Tracheostomy**	31600, 32551	0B110F4, 0B113F4
**Emergency intubation**	31500	
**ECMO**	33946, 33947, 33948, 33949, 33952, 33954, 33956, 33966, 33986	5A15223, 5A1522F, 5A1522G
**Dialysis**	90935, 90937, 90945, 90947, 90999, G0491, J0604	5A1D70Z, 5A1D80Z, 5A1D90Z)
**Ventricular septal defect**		I51.0, Q21.0, Q21.2

***** The presence of atrial fibrillation or flutter was determined by diagnosis code (ICD-10: I48.0, I48.1, I48.2, I48.4, I48.91). Patients with a code for atrial fibrillation or flutter at any point in the 30 days after SM that did not have a code prior to SM were considered to have a new diagnosis.

Table S2. Inpatient complications following septal myectomy by age group*.

Complications	Age < 65 (n=3,063)		Age ≥≥ 65 (n=2,261)	
Repeat septal myectomy	31	(1.0)	12	(0.5)
Cardiac arrest/CPR	49	(1.6)	76	(3.4)
TIA	16	(0.5)	23	(1.0)
Cerebral infarction	55	(1.8)	61	(2.7)
Ventricular fibrillation/tachycardia	419	(13.7)	194	(8.6)
Anoxic brain damage	7	(0.2)	1	(0.0)
Hemothorax	16	(0.5)	18	(0.8)
Cardiogenic/other/unspecified shock	248	(8.1)	215	(9.5)
Tracheostomy	48	(1.6)	44	(1.9)
Emergency intubation	46	(1.5)	54	(2.4)
ECMO	30	(1.0)	25	(1.1)
Acute kidney failure	330	(10.8)	400	(17.7)
New dialysis	42	(1.4)	36	(1.6)
Transfusion	145	(4.7)	144	(6.4)
Any atrial fibrillation/flutter	1151	(37.6)	1152	(51.0)
Cardioversion	121	(4.0)	114	(5.0)
New heart block and new pacemaker/ICD	259	(8.5)	267	(11.8)
New pacemaker/ICD	412	(13.5)	344	(15.2)

Complications	Age < 65 (n=3,063)		Age ≥≥ 65 (n=2,261)	
Coronary angiogram/PCI	113	(3.7)	87	(3.8)
Ventricular septal defect	110	(3.6)	63	(2.8)
Ventricular septal repair	29	(0.9)	20	(0.9)
No follow-up after SM**	16	(0.5)	32	(1.4)

**Data are n (%); **No diagnosis, procedure, surgery, or prescription claims within 30 days or 1 year after septal myectomy

Table S3. Distribution of predictors of CV hospitalization and new atrial fibrillation upon long term follow-up following SM.

	CV hospitalization				New Atrial Fibrillation			
**Characteristics**	**Yes**		**No**		**Yes**		**No**	
n	2381		2720		1294		3807	
Age, median (IQR)	63.0	(53.0, 71.0)	61.0	(51.0, 69.0)	65.0	(56.0, 72.0)	61.0	(51.0, 69.0)
Female, n (%)	1346	(56.5)	1370	(50.4)	697	(53.9)	2019	(53.0)
Prior diabetes, n (%)	393	(16.5)	325	(11.9)	195	(15.1)	523	(13.7)
Prior obesity, n (%)	493	(20.7)	464	(17.1)	236	(18.2)	721	(18.9)
Prior hypertension, n (%)	1463	(61.4)	1393	(51.2)	754	(58.3)	2102	(55.2)
Prior COPD, n (%)	241	(10.1)	120	(4.4)	124	(9.6)	237	(6.2)
Prior tobacco use, n (%)	494	(20.7)	495	(18.2)	227	(17.5)	762	(20.0)
Prior mitral valve disorder, n (%)	1033	(43.4)	1001	(36.8)	518	(40.0)	1516	(39.8)
Prior aortic valve disorder, n (%)	639	(26.8)	598	(22.0)	346	(26.7)	891	(23.4)
Prior tricuspid valve disorder, n (%)	118	(5.0)	97	(3.6)	46	(3.6)	169	(4.4)
Prior CKD, n (%)	233	(9.8)	160	(5.9)	121	(9.4)	272	(7.1)
Prior pacemaker, n (%)	266	(11.2)	153	(5.6)	105	(8.1)	314	(8.2)
Prior ICD, n (%)	117	(4.9)	50	(1.8)	37	(2.9)	130	(3.4)

Table S4. Predictors of new-onset atrial fibrillation following SM in long-term follow-up.

	Univariable Model		Full Model		Selected Model∗∗	
**Predictors**	**OR (95% CI)**	**P-value**	**aOR (95% CI)**	**P-value**	**aOR (95% CI)**	**P-value**
Age	1.03 (1.02, 1.03)	<0.0001	1.03 (1.02, 1.03)	**<0.0001**	1.03 (1.02, 1.03)	<0.0001
Female	1.04 (0.93, 1.16)	0.5105	0.91 (0.81, 1.01)	0.0855	0.91 (0.81, 1.02)	0.0957
COPD	1.49 (1.24, 1.79)	<0.0001	1.40 (1.15, 1.69)	**<0.0001**	1.42 (1.17, 1.71)	<0.0001
Tobacco use	0.89 (0.77, 1.03)	0.1229	0.84 (0.73, 0.98)	0.0243	0.85 (0.74, 0.99)	0.0332
CKD	1.39 (1.16, 1.68)	<0.0001	1.21 (1.00, 1.47)	**0.0455**	1.22 (1.01, 1.48)	0.0365
ICD	0.97 (0.79, 1.18)	0.7292	1.19 (0.97, 1.47)	0.1004	1.20 (0.97, 1.47)	0.0879
Pacemaker	0.83 (0.60, 1.15)	0.2656	0.78 (0.56, 1.10)	0.1565	0.78 (0.56, 1.09)	0.1438
Diabetes	1.16 (0.99, 1.35)	0.0596	1.05 (0.90, 1.23)	0.5437		
Obesity	1.04 (0.90, 1.19)	0.6135	1.10 (0.95, 1.27)	0.2138		
Hypertension	1.20 (1.07, 1.34)	0.0014	1.01 (0.89, 1.14)	0.8947		
Mitral valve disorder	1.05 (0.94, 1.18)	0.3567	1.04 (0.92, 1.16)	0.5362		
Aortic valve disorder	1.20 (1.06, 1.35)	0.0044	0.99 (0.87, 1.13)	0.9355		
Tricuspid valve disorder	0.87 (0.65, 1.17)	0.3656	0.80 (0.59, 1.09)	0.1573		

Abbreviations: OR=Odds Ratio, CI=Confidence Interval, aOR=adjusted Odds Ratio, COPD=Chronic Obstructive Pulmonary Disease ∗∗Model selected by backwards elimination based on AIC.

Figure S1. Flowchart summarizing patient selection process.

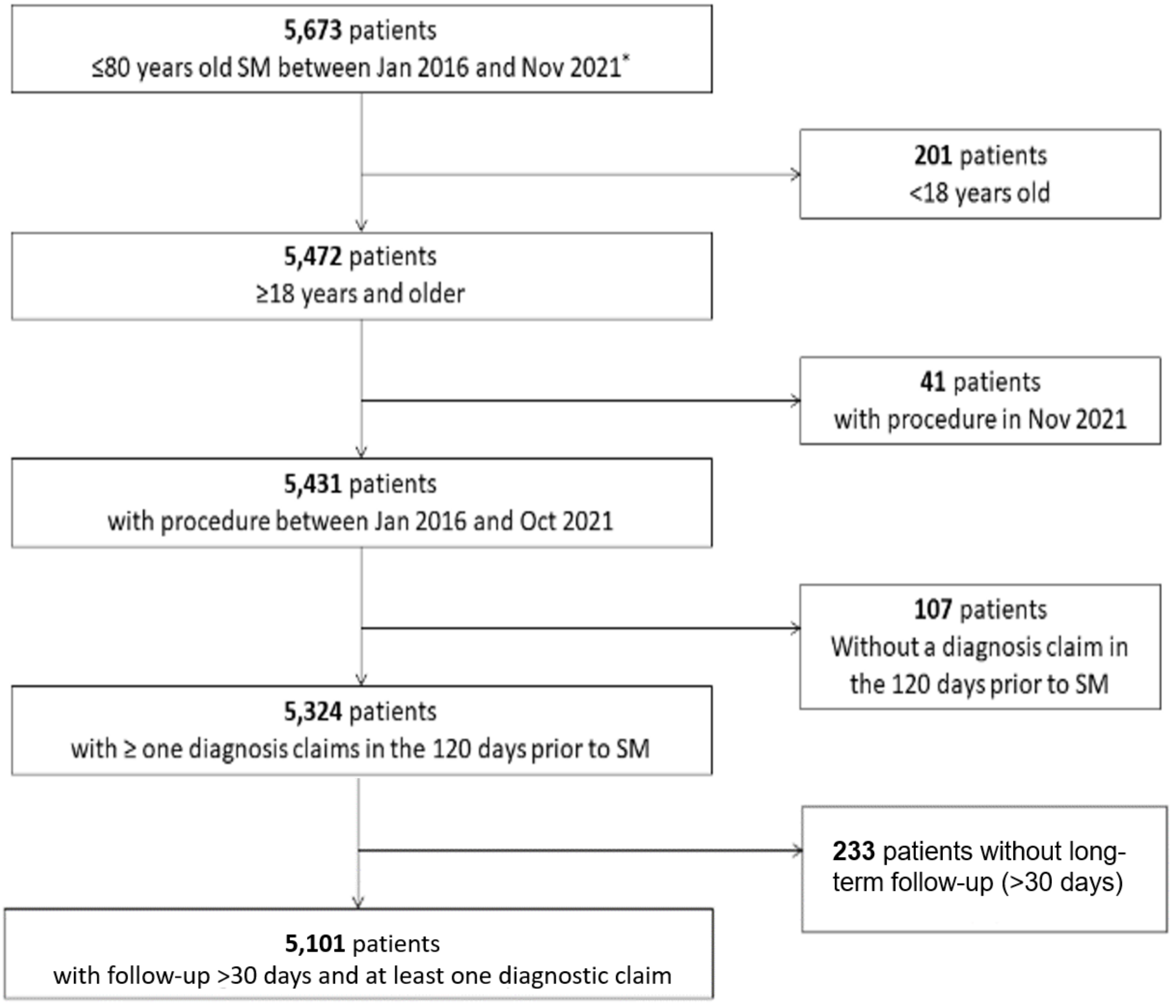

Supplement: Supplementary file 1 — Tables S1–S4 Figure S1 [file JAH3-14-e040655-s001.pdf]
